# Postoperative meningitis after spinal surgery: a review of 21 cases from 20,178 patients

**DOI:** 10.1186/1471-2334-14-220

**Published:** 2014-04-23

**Authors:** Tung-Yi Lin, Wen-Jer Chen, Ming-Kai Hsieh, Meng-Ling Lu, Tsung-Ting Tsai, Po-Liang Lai, Tsai-Sheng Fu, Chi-Chien Niu, Lih-Huei Chen

**Affiliations:** 1 Department of Orthopaedic Surgery, Spine Section, Chang Gung Memorial Hospital and Chang Gung University, No. 5, Fusing St., Guishan Township, Taoyuan 333, Taiwan

**Keywords:** Post spinal surgery, Postoperative meningitis, Complications

## Abstract

**Background:**

Postoperative bacterial meningitis is a rare complication of spinal surgery and is considered to be a complication related to intraoperative incidental durotomy. A high index of suspicion for meningitis is essential in patients who have the clinical triad of fever, neck stiffness and consciousness disturbance during the postoperative period. A delay in diagnosis or treatment can lead to morbidity and mortality. Due to the low incidence of postoperative meningitis, very few studies have reported this complication. The purpose of this study was to report the clinical features, laboratory evaluations, treatment course and prognosis of 21 patients with post spinal surgery meningitis.

**Methods:**

We retrospectively reviewed 21 patients (13 male, 8 female) with the diagnosis of postoperative meningitis after lumbar spinal surgery between January 2001 and Aug 2011. The median age of the patients was 67 years old (range 27 to 82 years) at the time of surgery. We recorded the preoperative diagnosis, operative methods, amount of drainage, clinical manifestations, laboratory evaluations, cerebrospinal fluid study, and infectious organisms. All patients diagnosed with postoperative meningitis received at least two weeks of antibiotic treatment. Clinical outcomes were assessed after at least two years of follow-up.

**Results:**

From January 2001 to August 2011, 20,178 spinal operations were performed in our institution, and 21 patients (0.10%) were diagnosed with postoperative meningitis. Eighteen patients (85.7%) had fever, 19 (90.5%) had neck stiffness, and 16 (76.2%) had consciousness disturbance. All patients had at least two of the classic triad. In addition, 9 patients (42.9%) had headache, 3 (14.3%) had focal neurological deficits, and 2 (9.5%) had seizure attacks. There was no mortality in this series. Postoperative meningitis showed no adverse effect on the results of spinal surgery after follow-up for at least two years.

**Conclusions:**

Postoperative meningitis is a rare complication after spinal lumbar surgery. A high index of suspicion for meningitis should be maintained in patients with the clinical triad of fever, neck stiffness, and consciousness disturbance after spinal surgery. Intraoperative incidental durotomy is the most important predictor. An early diagnosis and appropriate antibiotic treatment can lead to a good outcome.

## Background

Postoperative bacterial meningitis is a rare complication of spinal surgery [1] and is considered to be a complication related to incidental durotomy [2,3]. A high index of suspicion for meningitis is essential in patients who have the triad of fever, neck stiffness and consciousness disturbance during the postoperative period [4,5]. In addition, headache, seizure, and focal neurologic deficit may also occur [6]. A delayed diagnosis and treatment of meningitis may lead to adverse outcomes [7].

Postoperative bacterial meningitis may cause prolonged hospitalization, extended antibiotic treatment, and even mortality [8,9]. Due to the low incidence of postoperative meningitis, very few studies have reported this complication after spinal surgery. The purpose of this study was to report the clinical features, laboratory evaluations, treatment course and prognosis of 21 patients with post spinal surgery meningitis.

## Methods

After obtaining *institutional review board* approval, we retrospectively review 21 patients with the diagnosis of postoperative meningitis after lumbar spinal surgery in our institution between January 2001 and Aug 2011. All patients had presented acute clinical manifestations compatible with meningitis, confirmed by neurologists or infection specialists. Thirteen were male and 8 were female, with a median age of 67 years old (27–82) at the time of the surgery.

The preoperative diagnoses for spinal surgery included degenerative spondylolisthesis in 7 patients, degenerative lumbar scoliosis in 7 patients, lumbar spinal stenosis in 1 patient, herniated intervertebral disc in 4 patients, and segmental instability in 2 patients. The surgical methods included laminectomy in 17 patients, discectomy in 4 patients, transpedicle screw fixation with posterolateral fusion in 15 patients, and posterior or transforaminal interbody fusion in 10 patients. Cefazolin was routinely used as prophylaxis antibiotics which was given 30 to 60 minutes before incision. Postoperatively, cefazolin was administered for one day, however, for patients who received instrumentations or was American Society of Anesthesiologists (ASA) class III, cefazolin was given for 3 days. If incidental durotomy was observed intraoperatively, dural repair was performed immediately. Hemovac drainage was routinely used and draining was maintained for at least 3 days postoperatively.

If there was a suspicion of postoperative meningitis according to the clinical presentations, the work-up included complete blood count and differential count (CBC/DC), erythrocyte sedimentation rate (ESR), C-reactive protein (CRP), brain computed tomography (CT) and cerebrospinal fluid (CSF) studies. Empiric antibiotics were given immediately after the laboratory studies. In addition, neurologists and infection specialists were consulted for further recommendations. We recorded the clinical manifestations, postoperative Hemovac drainage, laboratory findings, CSF study results, brain CT findings, and infectious organisms. The clinical outcomes and complications were assessed perioperatively. The patients were followed up at 1, 3, 6 and 12 months after discharge, and then on an annual basis.

## Results

From January 2001 to August 2011, 20,178 spinal operations were performed at our institution, and 21 patients (0.10%) were diagnosed with postoperative meningitis. Fever was present in 18 patients (85.7%), neck stiffness in 19 patients (90.5%), and consciousness disturbance in 16 patients (76.2%). All of the 21 patients had at least two of the classic triad. In addition, 9 patients (42.9%) had headaches, 3 (14.3%) had focal neurological deficits, and 2 (9.5%) had seizure attacks. The mean onset of the first sign of meningitis (fever, neck stiffness, or consciousness change) was 7.9 ± 3.1 days (range 2 to 14 days) postoperatively.

Eleven (52.4%) patients had already been discharged after a smooth postoperative course, and then returned to our emergency department due to fever and/or consciousness disturbance. According to the medical records, 10 patients had incidental durotomy intraoperatively, and dural repair had been performed immediately. Another 11 patients had CSF leakage according to a massive amount of clear fluid drainage postoperatively. Postoperative meningitis had been suspected after one or more symptoms of the classic triad, and the other associated presentations developed later.

Laboratory data revealed leukocytosis (>10,000/uL) with mainly segmented cells in 14 patients (66.7%), and elevated CRP levels and ESR in all 21 patients. The median CRP level was 78 mg/L (range 28 to 178 mg/L), and the median ESR was 50 mm/h (range 42 to 78 mm/h), both of which were used as evaluation tools during the treatment course. Brain CT study showed negative findings in all of the patients. Electroencephalography (EEG ) was performed in 2 patients with seizure attacks as recommended by the neurologists.

After excluding the patients with bleeding tendency or suspected local wound infections, lumbar punctures were performed in 13 patients. One of the CSF cultures grew *Staphylococcus epidermidis* and the another grew *Pseudomonas aeruginosa*. In all cases, the CSF analysis revealed a white blood cell count above 1000/mL with a percentage of neutrophils greater than 50% or smear positive [10]. In addition, blood cultures was performed in all patients which were considered contraindication for lumbar puncture. The culture grew *Staphylococcus aureus* in 2 patients and coagulase-negative *Staphylococci,* which was not been fully identified, in 1 case. Another two patients presented with positive wound cultures (Table 1). All of the other patients without culture evidence were diagnosed with postoperative meningitis according to the clinical manifestations and laboratory data, which were confirmed by the neurologists and infection specialists.

**Table 1:** The diagnostic data of postoperative meningitis in 21 patients

**Age**	**Sex**	**Incidental Durotomy**	**Fever**	**Neck stiffness**	**Consciousness disturbance**	**Elevated in CRP & ESR**	**CSF analysis**	**Culture**
67	F	Y	Positive	Positive	Negative	Yes	Positive	Negative
82	M	N	Positive	Positive	Positive	Yes	Positive	Negative
65	F	N	Positive	Positive	Negative	Yes	Positive	Negative
27	M	Y	Positive	Positive	Positive	Yes	NP	NP
78	M	Y	Positive	Positive	Positive	Yes	Positive	Negative
65	F	N	Positive	Positive	Positive	Yes	NP	NP
73	F	Y	Positive	Positive	Negative	Yes	Positive	Negative
67	M	Y	Positive	Positive	Positive	Yes	NP	MSSA(B)
76	F	N	Positive	Positive	Positive	Yes	NP	NP
54	F	N	Positive	Positive	Positive	Yes	NP	CoNS (B)*
66	M	N	Negative	Positive	Positive	Yes	Positive	Negative
42	M	Y	Positive	Positive	Positive	Yes	NP	Staph.epidermidis (W)
69	M	Y	Positive	Positive	Negative	Yes	Positive	Negative
76	F	N	Positive	Positive	Negative	Yes	Positive	Negative
65	F	Y	Positive	Positive	Positive	Yes	NP	MRSA (B)
78	M	N	Positive	Negative	Positive	Yes	Positive	Negative
59	M	Y	Positive	Positive	Positive	Yes	Positive	Negative
75	M	N	Positive	Negative	Positive	Yes	Positive	Negative
74	M	N	Positive	Positive	Positive	Yes	NP	MSSA(W)
48	M	Y	Negative	Positive	Positive	Yes	Positive	Staph.epidermidis
29	M	N	Negative	Positive	Positive	Yes	Positive	Pseudo.aeruginosa

Y = incidental durotomy, N = no incidental durotomy, Positive = WBC >1000/mL with neutrophil greater than 50%, NP = not performed, B = blood culture, W = wound culture, CoNS = coagulase negative staphylococcus, MRSA = Methicillin-resistant Staphylococcus aureus, MSSA = Methicillin-sensitive Staphylococcus aureus *= the species not fully identified.

After the patients had been diagnosed with postoperative meningitis, treatment with two combined empiric antibiotics was given. Before 2004, a combination of oxacillin plus ceftriaxone was used in three cases. After 2004, the patients were treated with the regimen of vancomycin plus a third-generation cephalosporin such as ceftriaxone or ceftazidime for at least 2 weeks. The dosage was adjusted and modified according to the pathogen, clinical condition, and renal function of the patients.

After treatment, the fever subsided within 5 days in 17 patients (94.4%). All of the patients with neck stiffness improved during hospitalization, and 16 with consciousness disturbance recovered completely before discharge. Two patients who had underlying diabetes mellitus suffered from superficial wound infections, and 3 patients were complicated with pseudomeningocele and poor wound healing, in whom further surgery for dural repair was performed.

All 21 patients survived and recovered completely after at least 2 weeks of antibiotic treatment which was suggested by the neurologists and infection specialists. There was no mortality in this series. Seventeen of 21 cases had spinal implants , and there was no difference in the management and result of these 17 patients compared to other 4 patients. Postoperative meningitis showed no adverse effects on the result of spinal surgery after at least two years of follow-up, and no additional spinal revision surgery was performed except for dural repair.

## Discussion

Postoperative meningitis is a rare complication after spinal surgery, however it may cause severe sequelae including death. Twyman et al. reported an incidence of 0.18% after 2,180 spinal surgeries [1]. In our series, the incidence of postoperative meningitis is 0.10% (21 of 20,178 surgeries).

In the 21 patients with postoperative meningitis, fever was present in 85.7%, and most cases subsided within 5 days after empiric antibiotic treatment. Neck stiffness developed in 90.5% of the patients, and this should be a routine physical examination whenever incidental durotomy is recognized. Mental status was altered in 76.2% of the patients including lethargy, confusion, and coma. The incidence rates of these clinical presentations were similar to other large series of acute bacterial meningitis in adults [5,11]. All patients in our series had at least two of the classic triad. In addition, 42.9% of the patients had headache, 14.3% had focal neurological deficits, and 9.5% had seizure attacks. The incidence of headaches in our series was much lower than in other large series of acute bacterial meningitis in adults (79% to 94%) [7,11]. This may have been due to the use of postoperative analgesics.

Intraoperative incidental durotomy is not an uncommon complication in spinal surgery with a reported incidence of 0.3% to 13%, [12] and postoperative meningitis is likely to occur in patients with intraoperative incidental durotomy. In our series, all of the patients had either CSF leakage noted during the operation or from massive amounts of clear fluid drainage postoperatively. Jankowitz et al. [13] retrospectively analyzed 4,835 lumbar operations, and found that 547 (11.3%) patients had intraoperative incidental durotomy with a frequency of 9% at the primary surgery and 21% at the revision. Morris et al. [14] reported two cases of bacterial meningitis in two traumatic dural laceration patients who underwent posterior instrumentation with pedicle screws. Both patients had good results, and the authors concluded that early recognition of meningitis plus timely treatment may lead to a favorable outcome. The occurrence of pseudomeningocele is a possible sequelae of a dural tear and has been reported to be related to imperfect suture of the dura or fascia, and it may require surgical intervention [15,16]. In our series, pseudomeningocele was noted in 3 cases, and all of them required surgery for dural repair.

The mean onset of initial symptoms or signs related to meningitis was 7.9 ± 3.1 days (range 2 to 14 days) postoperatively. Eleven patients were discharged after a smooth postoperative course, but returned to our emergency department due to complaints related to meningitis, such as fever, consciousness disturbance, seizure, or severe headache. This finding should remind surgeons that postoperative meningitis may present as a delayed complication after spinal surgery, even in the patients who are discharged after a smooth postoperative course. Da Costa et al. [17] reported a case of postoperative meningitis with an onset time of 5 years after removal of the spinal instrumentation for scoliosis. The patients suffered from repeated episodes of meningitis because an infected cyst in the bone cavity where the previous instrumentation were situated communicated with the intradural space. He was free from meningitis after surgical intervention for dural closure and cyst debridement. As mentioned previously, a high index of suspicion is necessary for the patients who have the classic triad of meningitis after spinal surgery.

Antibiotic treatment should be initiated immediately after a lumbar puncture is performed or a blood culture obtained [18]. The administration should not be unduly delayed particularly if imaging is being performed before lumbar puncture. A delay in antibiotic treatment can occur when the presentation is atypical, when waiting for imaging studies, or even in neglected cases of meningitis. This delay should be avoided to prevent adverse outcomes such as mortality and unrecoverable neurological deficits [7,19].

Appropriate antibiotics should have the ability to penetrate the blood–brain barrier and cover the most likely pathogens [20]. In addition, in order to obtain adequate concentrations in the CSF, specific dosages should be given to treat bacterial meningitis [21]. In most cases, a combination of two antibiotics is suggested for at least two weeks with the regimen of vancomycin at a dosage of 1 g every 8 to 12 hours plus a third generation cephalosporin (for example, ceftriaxone 2 g every 12 hours) [18,22]. However, the regimen can also be shifted to vancomycin plus ceftazidime as this has a better activity against *Pseudomonas aeruginosa* or vancomycin plus meropenem which has a better activity against anaerobic pathogens and is more suitable for immunocompromised patients.

A lumbar puncture should be performed for every patient with suspected meningitis if there are no contraindications. Blood cultures are also suggested for those who develop a fever. Brain CT is suggested to rule out other causes of consciousness disturbance, however imaging studies should not delay the antibiotic treatment. In addition, electrolyte balance and fluid management with the use of intravenous hydration has been shown to be beneficial for patients with meningitis [23]. Recommendations from neurologists and infection specialists may also be useful in the diagnosis and treatment.

There are several limitations to this study. First, not all of the patients had a definite diagnosis of bacterial meningitis according to the CSF cultures or analysis. However, for patients with a bleeding tendency or those who are at risk of local infection, a lumbar puncture should be avoided. Without the results of a CSF culture, the diagnosis of meningitis was made according to clinical presentations, laboratory data, and blood cultures, and confirmed by neurologists and infection specialists. The use of perioperative antibiotics is routine practice in spinal surgery, and this may result in a negative CSF bacterial culture postoperatively. Second, the rare incidence of postoperative meningitis resulted in a limited number of cases.

## Conclusion

This study showed that postoperative meningitis is a rare complication after spinal lumbar surgery. A high index of suspicion for meningitis is essential in patients who have the clinical triad of fever, neck stiffness, and consciousness disturbance after spinal surgery. Intraoperative incidental durotomy is the most important predictor. Early diagnosis and appropriate antibiotic treatment for at least two weeks can lead to a good outcome.

### Ethic statement

Protocol No: CGMF IRB No.: 101-3850B from Institutional Review Board Chang Cung Medical Foundation.

## Competing interest

The authors have declared that they have no competing interest.

No funds were received in support of this work. No potential benefits in any form from a commercial party related directly or indirectly to the subject of this manuscript.

## Authors’ contributions

T-YL and W-JC drafted the manuscript and design of the study. M-KH and M-LL analysis and interpretation of the data. T-TT, P-LL, and T-SF participated in the data collecting. C-CN and L-HC conceived of this study and participated in the design. All authors read and approved the final manuscript.

## Pre-publication history

The pre-publication history for this paper can be accessed here:

http://www.biomedcentral.com/1471-2334/14/220/prepub
